# Identification of CD105 (endoglin) as novel risk marker in CLL

**DOI:** 10.1007/s00277-022-04756-4

**Published:** 2022-01-19

**Authors:** Sarah M. Greiner, Melanie Märklin, Samuel Holzmayer, Kübra Kaban, Sophie Meyer, Clemens Hinterleitner, Claudia Tandler, Ilona Hagelstein, Gundram Jung, Helmut R. Salih, Jonas S. Heitmann, Joseph Kauer

**Affiliations:** 1 Clinical Collaboration Unit Translational Immunology, German Cancer Consortium (DKTK), Department of Internal Medicine, University Hospital Tübingen, Otfried-Müller-Str. 10, 72076 Tübingen, Germany; 2 Cluster of Excellence iFIT (EXC 2180) “Image-Guided and Functionally Instructed Tumor Therapies”, Eberhard Karls University, Tübingen, Germany; 3 Department of Medical Oncology & Pneumology, University Hospital Tübingen, Tübingen, Germany; 4 Interfaculty Institute for Cell Biology, Department of Immunology, German Cancer Consortium (DKTK) and German Cancer Research Center (DKFZ), Partner Site Tübingen, University of Tübingen, Tübingen, Germany; 5 Department of Internal Medicine V. Hematology, Oncology and Rheumatology, University Hospital Heidelberg, Heidelberg, Germany

**Keywords:** Chronic lymphocytic leukemia, Risk assessment, Flow cytometry

## Abstract

Several genetic and clinical markers are established as prognostic factors in chronic lymphocytic leukemia (CLL). However, additional markers are needed for risk stratification. Flow cytometric analysis is a mainstay of CLL diagnostics, thus identification of novel prognostic surface markers can improve risk assessment without increasing burden for patients and physicians. Furthermore, surface molecules preferentially expressed in high-risk cases could serve as therapeutic targets for immunotherapy. CD105 (endoglin) is a TGF-beta coreceptor and activates endothelial cells in healthy tissues and cancer. In addition, it is expressed on healthy hematopoietic precursors as well as lymphoid and myeloid leukemias. In acute myeloid leukemia (AML), a CD105 antibody is successfully applied in clinical studies. In CLL, mRNA expression of the CD105 gene ENG reportedly correlates with other risk factors but failed to show significant correlation with overall survival. However, CD105 protein expression in CLL has never been studied. We here analyzed CD105 surface expression on CLL cells from 71 patients by flow cytometry and report for the first time that substantial levels of CD105 are detectable on CLL cells in 70.4% of patients. Using receiver operating characteristics, we established a cutoff of 5.99% positive cells to distinguish between low and high CD105 levels, the latter correlating with decreased time to first treatment and overall survival. High CD105 expression further correlates with CD38 expression. Our study identified membrane expression of CD105 as a potential risk marker and therapeutic target in high-risk CLL. However, multivariant analyses of large cohorts should be performed in confirmatory studies.

**Supplementary Information:**

The online version contains supplementary material available at 10.1007/s00277-022-04756-4.

## Introduction

Chronic lymphocytic leukemia (CLL) is a malignant disorder with a highly variable course and prognosis [1]. Established risk factors involve parameters obtained upon clinical assessment or genetic analyses [2–5]. Immunophenotyping of peripheral blood CLL cells is a widely available cornerstone of CLL diagnosis [6, 7], and some markers obtained by flow cytometric analysis like CD38 and CD49d are well established to predict outcome [8–11].

CD105 (endoglin) is an accessory molecule within the transforming growth factor (TGF) beta receptor signaling complex [12, 13] and mediates activation of endothelial cells [14–16]. It is expressed on the neovasculature of many solid tumor entities where expression correlates with poor prognosis [17–22]. In the hematopoietic system, CD105 expression has been reported for healthy hematopoietic stem cells [23–25] and neoplasias like myelodysplastic syndrome (MDS), acute lymphoblastic leukemia (ALL), and acute myeloid leukemia (AML) [26, 27]. In the latter, CD105 expression correlates with poor outcome [28] and is increasingly evaluated as therapeutic target. In CLL, it has been reported that mRNA levels of the CD105 gene ENG associate with other risk markers, but failed to show significant correlation with overall survival [29]. Plasma levels of soluble CD105 display prognostic significance [30]. The prognostic impact of CD105 surface expression remains unclear. Here we report on CD105 levels on CLL cells and their association with disease outcome in 71 patients.

## Methods

### Patient samples

Peripheral blood was obtained from 71 patients with CLL from 2007 to 2019 after confirmed diagnosis. Median time between diagnosis and sample acquisition was 4 years (interquartile range 2–9 years). All experiments have been approved by the appropriate ethics committee (Ethics Committee of the University of Tübingen vote 13/2007 V) and have therefore been performed in accordance with the ethical standards laid down in the 1964 Declaration of Helsinki and its later amendments. Informed consent was obtained from all patients prior to initiation of experiments. Peripheral blood mononuclear cells (PBMC) were isolated using density gradient centrifugation using Biocoll Solution (Biochrom AG, Berlin, Germany) and thereafter stored in liquid nitrogen. Mean observational time for patients was 114 months (95% CI 96.8–132.6 months). Diagnosis was based on the iwCLL guideline [31]. Classification of CLL cases was performed according to the recent Rai and Binet classification systems [2–5]. Cytogenetic analyses were performed with standardized methods at the medical care center (MVZ) Dortmund laboratory.

### Flow cytometry

PBMC of CLL patients were incubated with human IgG (Sigma-Aldrich, St. Louis, MO) solution at 10 μg/ml for 30 min at room temperature prior to antibody staining. Unconjugated anti-CD105 mAb (clone K-ro23) or isotype control (clone MPC-11, BD Biosciences, Heidelberg, Germany) was added at 5 nM, followed by a goat-anti-mouse PE conjugate (Jackson Immunoresearch, West Grove, USA, 1:100). CLL cells were identified by staining for CD5 and CD19. Only viable cells were included based on 7-AAD (BioLegend, San Diego, USA) negativity. Fluorescence-antibody conjugates (CD5-APC and CD19-FITC from BioLegend) were used in 1:50–1:100 dilutions. Measurements were conducted with a FACSCanto II (BD Biosciences). Data analysis was performed using FlowJo_V10 software (FlowJo LCC, Ashland, OR). Specific fluorescence intensity (SFI) levels were calculated by dividing median fluorescence intensities obtained with anti-CD105 mAb by median fluorescence intensities obtained with the respective fluorescence-minus-one (FMO) control. Expression was considered positive in case of SFI ≥ 1.5. A technical validation of our staining method using primary CLL cells is provided as Supplementary Figure S1.

### Statistical analysis

Data are displayed as box plots with min/max whiskers or as individual dots according to mean values. For statistical analysis, Pearson-chi^2^, two-sided Fisher’s exact test, Mann–Whitney *U* test, or Kruskal–Wallis test was used to compare individual groups. If applicable, adjustment for multiple comparison was done. Correlation analysis was done by Spearman correlation. Distribution of overall survival (OS) was calculated using the Kaplan–Meier method. Log-rank test was performed to test for difference of survival between groups. For predictive cutoff value estimation, we subgrouped CD105 SFI with respect to corresponding OS times. Statistical analyses were conducted using GraphPad Prism 8.4.0 and JMP® Pro (SAS Institute Inc., version 14.2) software. *P* values < 0.05 were considered statistically significant.

## Results

### Clinical characteristics of the patient cohort

For assessment of CD105 expression, CLL samples from 71 patients were analyzed. The clinical characteristics of the patients are shown in Table 1. The age ranged from 36 to 80 years, with a median age of 62. The male to female ratio was 1.63:1. The majority of patients presented with Binet stage A at diagnosis (*n* = 49), 14 with stage B, and 5 with stage C. Rai stage 0 was diagnosed in 24 patients, stages I–II in 29 patients, and stages III–IV in 6 patients. *IGHV* mutational status was determined in 30 patients, with equal distribution of mutated and unmutated genes. Expression of prognostic markers such as CD38 expression < 20% was observed in 47 patients, 20–29% in 4 patients, and ≥ 30% in 11 patients. Three out of 35 tested patients were identified as *TP53*-mutated. Metaphase cytogenetic analysis was performed for 23 patients, which were subsequently grouped according to prognosis: favorable (12 trisomy, del 13q, normal karyotype; *n* = 15) and poor (del17p, del11q; *n* = 8).

**Table 1:** Patient characteristics

	Number of patients (%) (total number of patients *n* = 71)
Sex	
Male	44 (62)
Female	27 (38)
Median age at diagnosis (years)	62 (range 36–80)
Binet stage at sample acquisition	
A	30 (43)
B	22 (32)
C	17 (25)
n/a	2
Rai stage at sample acquisition	
0	9 (13)
I–II	39 (57)
III–IV	21 (30)
n/a	2
*IgHV* mutational status	
Mutated	15 (50)
Unmutated	15 (50)
n/a	41
CD38 expression	
< 20%	47 (76)
20–29%	4 (6)
≥ 30%	11 (18)
n/a	9
Cytogenetic risk*	
Favorable	15 (65)
Poor	8 (35)
n/a	48
*TP53* mutation	
Positive	3 (9)
Negative	32 (91)
n/a	36
Laboratory parameters	
Lymphocyte count (1/μl)	59,150 (range 7795–320,330)
Hb (g/dl)	13 (range 7.3–15.9)
Plt (1000/μl)	175 (range 18–418)
β-2 Microglobulin (mg/l)	3.9 (range 1.7–16.8)

*Hb*, hemoglobin; *plt*, thrombocytes; *n/a*, not available. *Classical cytogenetic analysis were performed and patients were grouped accordingly: favorable: 12 trisomy, del 13q, normal karyotype; poor: del17p, del11q

### CD105 is heterogeneously expressed on peripheral CLL cells

**Fig. 1:**
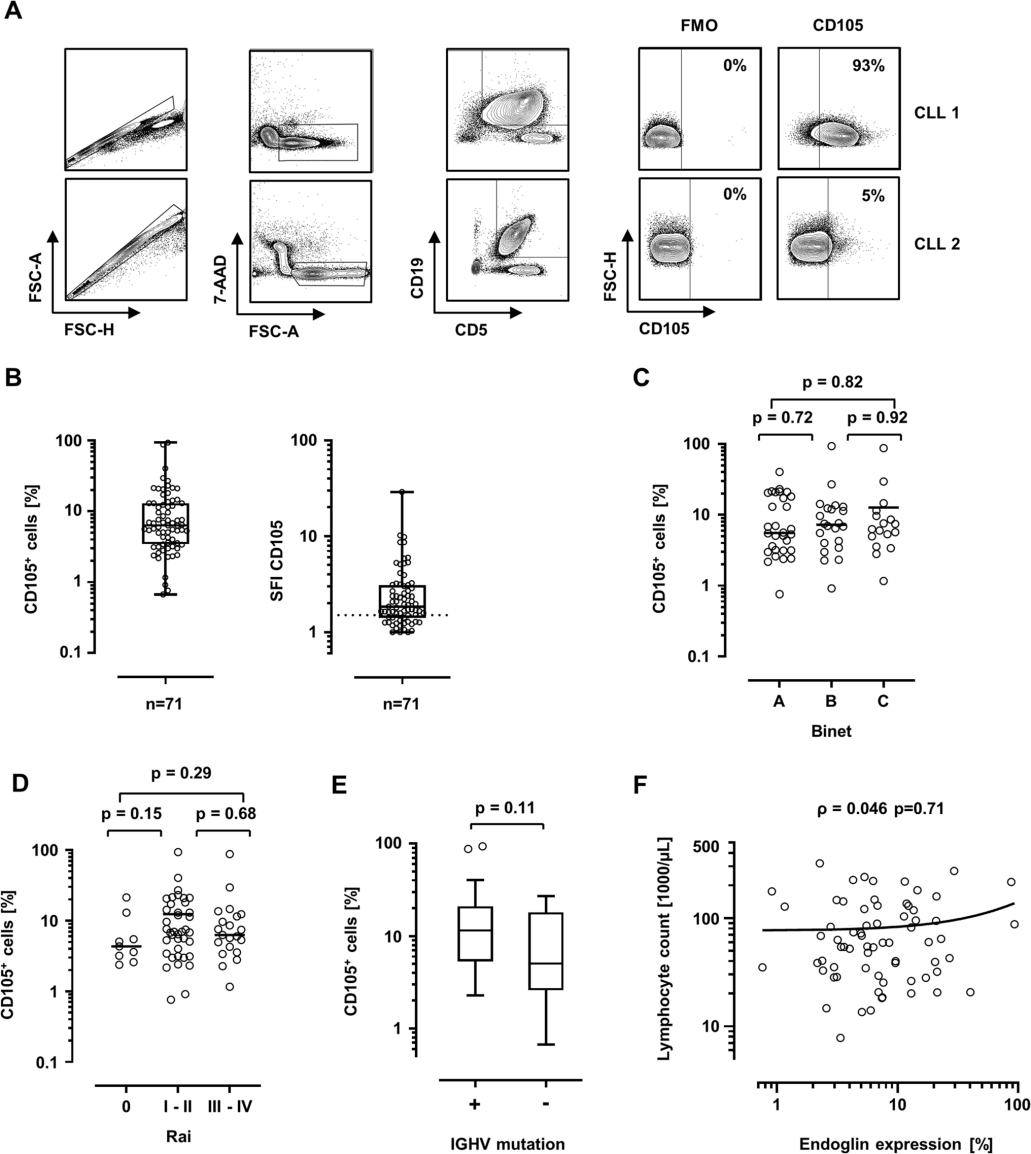
CD105 expression on CLL cells analyzed by flow cytometry. **A** An exemplary gating strategy is pictured: singlets, viable (7-AAD^−^), CD5^+^CD19^+^ CLL cells, CD105 expression versus fluorescence-minus-one (FMO) control. **B** CD105 expression on CLL cells of patients (*n* = 71) are shown as percentage of CD105-positive cells and specific fluorescence intensity (SFI) levels. SFI levels above 1.5 were considered positive (dotted line). (box plot with min/max whiskers). **C** Distribution of CD105 expression (% positive cells) according to Binet stage at sample acquisition is shown (single values, mean, Kruskal–Wallis test, Dunns’ correction). **D** CD105 expression (% positive cells) according to Rai stage at sample acquisition is depicted (single values, median, Kruskal–Wallis test, Dunns’ correction). **E** CD105 expression levels (% positive cells) according to *IGHV* mutational status (boxplots with Tukey whiskers, Mann–Whitney *U* test). **F** Correlation of peripheral blood lymphocyte count and CD105 expression. (single values, Spearman correlation, simple linear regression)

**Fig. 2:**
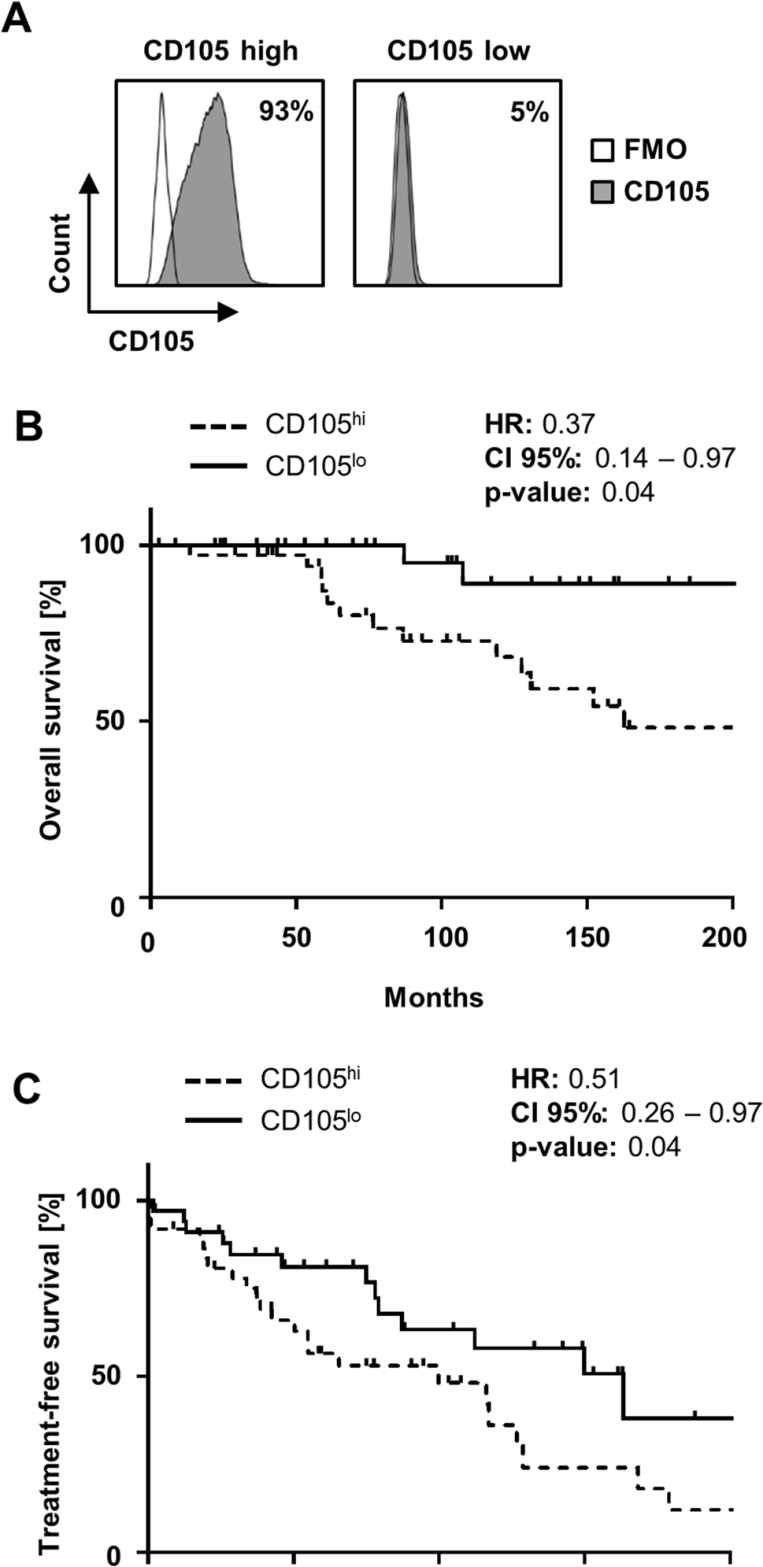
The impact of CD105 expression on survival in CLL. **A** Specific binding of the CD105 antibody K-ro23 (shaded peaks) and a fluorescence-minus-one (FMO) control (open peaks) at 5 nM on CLL cells from exemplary patients of the CD105^hi^ and CD105^lo^ groups was analyzed by flow cytometry. **B** Overall survival (OS) according to CD105^lo^ and CD105^hi^ expression is presented (Kaplan–Meier analysis, log-rank test). Median OS in CD105^hi^ cases was 162.7 months (dotted line; log-rank test). **C** Time to first treatment (TTFT) after sample acquisition according to CD105^lo^ and CD105^hi^ expression is shown (Kaplan–Meier analysis, log-rank test). In CD105^hi^, median TTFT was 29.4 months (dotted line)

As initial step, CD105 expression was determined on CD5^+^CD19^+^ CLL cells by flow cytometry. An exemplary gating strategy is depicted in Fig. 1A. SFI expression levels and percentages of CD105^+^ cells were heterogeneously distributed among the cohort and ranged from 1.0 to 28.9 and < 1 to 93.3% CD105^+^ cells, respectively (Fig. 1B). The median SFI within the cohort was 1.85. Considering a CD105 expression SFI of 1.5 as positive, 50 out of 71 patients (70.4%) were defined as CD105-positive. No significant difference was noted for CD105 levels according to different Binet and Rai stages at sample acquisition (Fig. 1C, D). A tendency to higher CD105 levels was observed for Rai stages I–II versus Rai stage 0, but failed to reach statistical significance (*p* = 0.15). *IGHV*-mutated cases exhibited higher CD105 levels without showing statistical significance (*p* = 0.11) (Fig. 1E). Lymphocyte counts in the peripheral blood did not correlate with CD105 expression levels (*ρ* = 0.046, *p* = 0.71, Fig. 1F). Using Youden index, we calculated a cutoff of 5.99% positive cells by using receiver operating characteristics (ROC) analysis to distinguish between CD105^hi^ and CD105^lo^ cases, the latter group also comprising CD105-negative cases. Figure 2A shows exemplary histogram plots of CD105^hi^ and CD105^lo^ patients.

### CD105 expression is predictive of outcome in CLL

In order to further evaluate the association of CD105 expression and survival in CLL and to confirm the predictive value of our calculated cutoff, Kaplan–Meier analyses were performed. When grouped according to CD105 expression, CD105^hi^ cases exhibited a significantly shorter overall survival (OS) (Fig. 2B, p = 0.04). Median OS in CD105^hi^ cases was 162.7 months, whereas median OS in CD105^lo^ was not reached. In addition, median time to first treatment (TTFT) after sample acquisition was found to be significantly shorter in CD105^hi^ patients (Fig. 2C) and was reached after 29.4 months, whereas in CD105^lo^, median TTFT was not reached (*p* = 0.04).

Next, the association of CD105 expression and established patient characteristics as well as common prognostic markers was studied using our cutoff value (Table 2). CD105^hi^ cases showed an overrepresentation of poor risk cytogenetics, but failed to reach statistical significance (*p* = 0.06). Furthermore, higher CD38 expression was observed in CD105^hi^ cases (*p* = 0.04). No significant differences were observed for Binet and Rai stages, neither at initial diagnosis nor at the time of sample acquisition. Neither *TP53* mutational status nor *IGHV* mutational status nor *TP53* mutations correlated with CD105 expression levels. However, molecular genetics and FISH data were not available for all patients.

**Table 2:** Patient characteristics according to CD105^lo^ and CD105^hi^

	Number of patients (%)		*p*-value
	CD105^lo^ (≤ 5.99% positive cells) *n* = 34	CD105^hi^ (> 5.99% positive cells) *n* = 37	
Sex			
Male	18 (53)	26 (70)	0.15^┘^
Female	16 (47)	11 (30)	
Median age at diagnosis (years)	61 (range 38–80)	67 (range 36–79)	0.59^†^
Binet stage at sample acquisition			
A	16 (50)	14 (38)	0.48^‡^
B	8 (25)	14 (38)	
C	8 (25)	9 (24)	
n/a	2	0	
Rai stage at sample acquisition			
0	7 (22)	2 (5)	0.31^‡^
I–II	15 (47)	24 (65)	
III–IV	10 (31)	11 (30)	
n/a	2	0	
*IgHV* mutational status			
Mutated	9 (64)	6 (37)	0.27^┘^
Unmutated	5 (36)	10 (63)	
n/a	20	21	
CD38 expression			
< 20%	23 (88)	24 (67)	0.04^‡^
20–29%	0 (0)	4 (11)	
≥ 30%	3 (12)	8 (22)	
n/a	8	1	
Cytogenetic risk			
Favorable*	6 (100)	9 (53)	0.06^┘^
Poor*	0 (0)	8 (47)	
n/a	28	20	
*TP53* mutation			
Negative	12 (100)	20 (87)	0.54^┘^
Positive	0 (0)	3 (13)	
n/a	22	14	
Laboratory parameters			
Lymphocyte count (1/μl)	54,355 (range 7795–320,330)	59,900 (range 18,234–272,583)	0.71^†^
Hb (g/dl)	12.5 (range 9.4–15.5)	13.1 (range 7.3–15.9)	0.34^†^
Plt (1000/μl)	160 (range 18–294)	184 (range 21–418)	0.34^†^
β-2 Microglobulin (mg/l)	3.9 (range 2.2–9.7)	3.8 (range 1.7–16.8)	1^†^

*Hb*, hemoglobin; *Plt*, thrombocytes; *n/a*, not available. Statistical analysis with ^┘^two-sided Fisher’s exact test, ^‡^Pearson-chi^2^, and ^†^Mann–Whitney *U* test. *Classical cytogenetic analysis were performed and patients were grouped accordingly: favorable: 12 trisomy, del 13q, normal karyotype; poor: del17p, del11q

## Discussion

The TGF-beta type III receptor CD105 is an important activator of endothelial cells in healthy and malignant tissues [13, 32]. Furthermore, it is involved in self-renewal of hematopoietic precursor cells [23, 24]. CD105 has also been described to be expressed on malignant cells in MDS and ALL [26, 27]. In AML, CD105 expression reportedly correlates with poor outcome, which held true even after hematopoietic stem cell transplantation [26, 28, 33]. Whereas distinct mRNA expression of the CD105 gene ENG was found in CLL cells [29], surface expression of CD105 in CLL has so far never been studied, and to our knowledge, we thus here provide the first report on CD105 surface expression in CLL.

Using flow cytometric analysis of CLL cells, we found CD105 positivity in about 70% of all patients. Similar prevalence was reported for AML, where 37–92% of cases were described as positive [26, 28] as well as for ALL with about 68% reportedly positive cases [26]. Regarding expression levels (SFI), however, AML blasts in general exhibited higher amounts of protein (maximum SFI 65.2) compared to CLL cells (maximum SFI 28.9) as assessed by us using the same antibody for detection [28]. Interestingly, healthy B cells, in contrast to leukemic B cells in CLL, do not express CD105 [28], which indicates that CD105 is acquired during and may play a role in malignant transformation.

Flow cytometric analysis of peripheral blood leukemic cells is a method that is easy to perform and increasingly used for diagnostic purposes. In CLL, identification of CD19^+^CD5^+^ cells within the peripheral blood constitutes the cornerstone of diagnostics. Flow cytometry is broadly applicable and established, and results are rapidly available at relatively low costs. Immunophenotyping of CLL cells not only reliably facilitates diagnosis, but also provides prognostic information by analysis of established markers such as CD38 and CD49d for risk stratification. The presently used panels can easily be adjusted by inclusion of additional prognostic surface markers.

Clinical evaluation according to Rai and Binet stages is long established to be prognostically relevant. However, clinical assessment is often vague and does not fully account for the broad range of disease courses in CLL. While some patients never exhibit symptoms and die eventually from causes unrelated to CLL, others display a steadily progressive disease. Therefore, it is of utmost importance to avoid both insufficient and excessive treatments.

CD105 mRNA expression in CLL reportedly correlates with shorter time to first treatment and a more aggressive clinical course of the disease. We found a significant correlation of CD105^hi^ expression with both shorter OS and TTFT. This is in line with the weak association of other risk markers and high CD105 mRNA levels described in the literature [29]. The strong correlation between CD105 expression and worse outcome in CLL observed in our study suggests that CD105 accelerates disease progression. However, the functional role of CD105 protein in CLL is yet not understood and should be subject to further investigation. Since the prognostic marker CD49d plays an important role in homing of CLL cells to secondary lymphoid organs [11], CD105 might be linked to interaction with endothelial cells and homing of CLL cells. Furthermore, CLL cells reportedly are capable of exerting immunosuppressive activity. For instance, B regulatory cell-like CLL cells secrete TGF-β and thereby transform naïve T helper cells into T regulatory cells [34]. It is tempting to speculate that the TGF-ß coreceptor CD105 may also be involved in immunosuppression in CLL.

TGF-ß inhibits proliferation in healthy B cells and CLL cells. The latter can overcome this disadvantage by lowering the expression of TGF-ß receptors, thus reducing its antiproliferative effect [35, 36]. It seems possible that CD105 is involved in the process of evading TGF-ß effects, as it was described to modulate TGF-ß signaling, e.g., in T cells [37, 38]. This could at least partially explain the correlation of high CD105 expression on CLL cells and worse outcome.

The high prevalence and expression levels of CD105 as observed in adverse cases indicate that CD105 may constitute an attractive therapeutic target in CLL. Since an anti-CD105 antibody termed TRC105 is already evaluated in clinical trials in other disease entities and so far exhibits a promising safety profile [39–41], this antibody could readily be tested in CLL. Therapeutic effects could rely on both the blockade of CD105 signaling and recruitment of immune effector cells alike and ultimately improve and complement established regimens.

However, our study exhibits certain limitations. These include the low number of patients, a long interval between first diagnosis and CD105 testing, and missing data on prognostic factors such as *IGHV* mutational status and cytogenetics, thus precluding multivariate analysis. The association of CD105 expression and worse outcome in CLL should be further analyzed in cohorts eligible for multivariate analyses. Furthermore, indirect staining of CLL cells with our proprietary antibody clone might not be widely available, thus limiting the reproducibility of our study. The prognostic relevance of CD105 in CLL should be confirmed by flow cytometry data obtained with direct staining methods.

In summary, we first described CD105 expression on the surface of CLL cells and showed a strong correlation with worse outcome in CLL. There was an association of high CD105 expression with shorter TTFT and OS, possibly because of higher proportion of cases with unfavorable biological prognosis. CD105 might serve as a novel prognostic marker in CLL but confirmatory studies in larger patient cohorts and including multivariable analysis are necessary.

## Supplementary Information

Below is the link to the electronic supplementary material.

Supplementary file1 (PDF 175 KB)

## Abbreviations

ALL: Acute lymphoblastic leukemia
AML: Acute myeloid leukemia
CLL: Chronic lymphocytic leukemia
MDS: Myelodysplastic syndrome
OS: Overall survival
ROC: Receiver operating characteristics
SFI: Specific fluorescence index
TGF: Transforming growth factor
TTFT: Time to first treatment
