# Supplementary material for: Identification of CD105 (endoglin) as novel risk marker in CLL
Source: Ann Hematol. 2022 Jan 19;101(4):773–80. doi: 10.1007/s00277-022-04756-4 (PMC8913466; doi:10.1007/s00277-022-04756-4)
Supplement: Supplementary file 1 — Supplementary file1 (PDF 175 KB) [file 277_2022_4756_MOESM1_ESM.pdf]

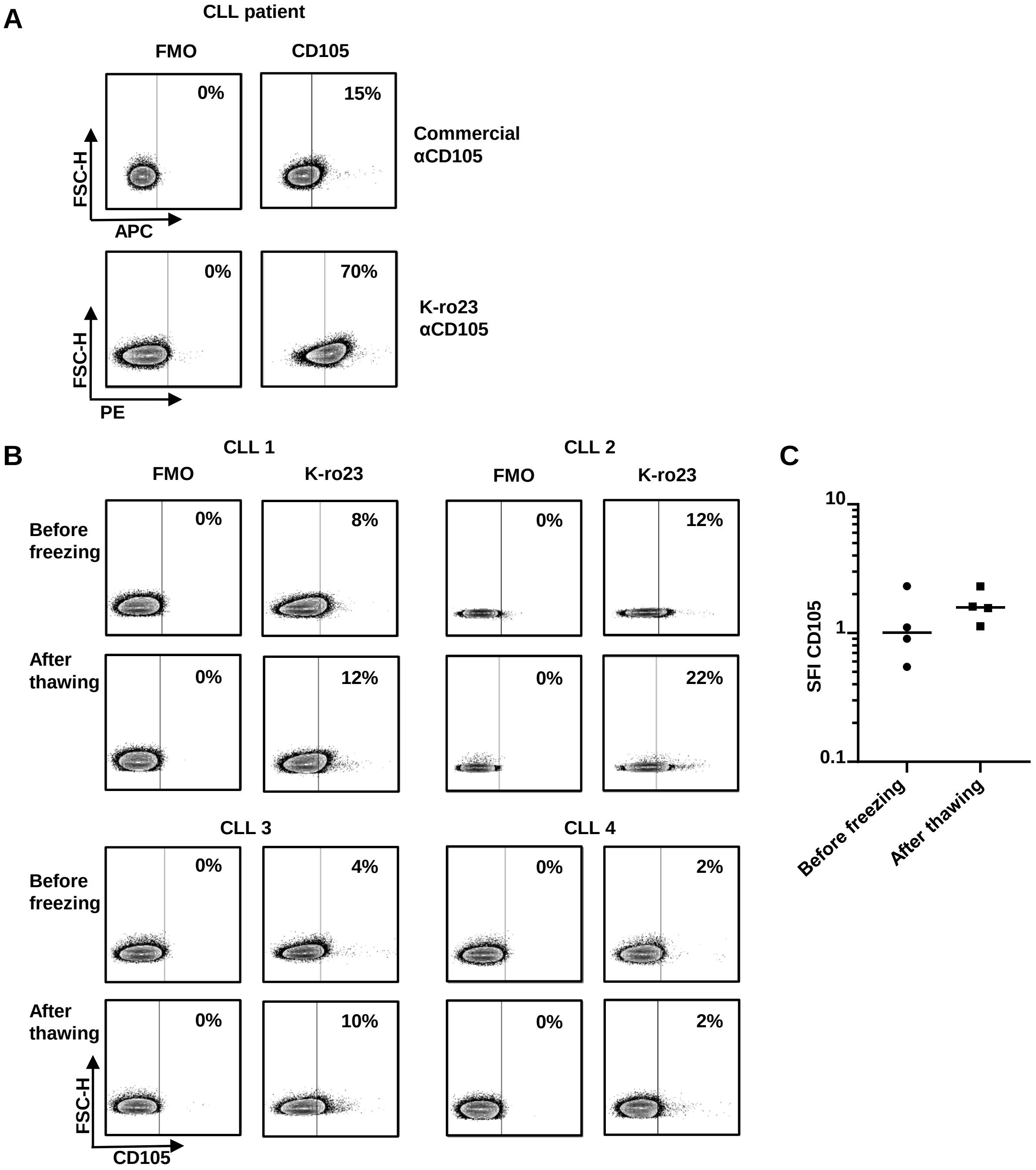

Supplementary Figure S1: CD105 expression on primary CLL cells assessed by indirect and direct flow cytometric staining

A CD105 expression on primary CLL cells stained with the directly labeled anti-CD105-APC antibody (top) (Biolegend, clone 43A3, Cat: 323208) compared to anti-CD105 antibody K-ro23 (bottom). **B+C** Four different primary CLL samples were analyzed for CD105 expression before freezing and after thawing. CD105 positivity and SFI levels were defined according to the FMO, respectively 2nd mAb controls.
